# *Hermetia illucens* larvae as a potential dietary protein source altered the microbiota and modulated mucosal immune status in the colon of finishing pigs

**DOI:** 10.1186/s40104-019-0358-1

**Published:** 2019-06-19

**Authors:** Miao Yu, Zhenming Li, Weidong Chen, Ting Rong, Gang Wang, Xianyong Ma

**Affiliations:** 1 Institute of Animal Science, Guangdong Academy of Agricultural Sciences, Guangzhou, 510640 Guangdong People’s Republic of China; 2 State Key Laboratory of Livestock and Poultry Breeding, Guangzhou, 510640 Guangdong People’s Republic of China; 3 Key Laboratory of Animal Nutrition and Feed Science in South China, Ministry of Agriculture; Guangdong Public Laboratory of Animal Breeding and Nutrition, Guangzhou, 510640 Guangdong People’s Republic of China; 4 Guangdong Engineering Technology Research Center of animal Meat quality and Safety Control and Evaluation, Guangzhou, 510640 Guangdong People’s Republic of China

**Keywords:** Colon, Finishing pigs, *Hermetia illucens* larvae, Immune status, Microbial metabolites, Microbiota

## Abstract

**Background:**

Insects, such as *Hermetia illucens* larvae, are rich in chitin and proteins, and represent a suitable feed ingredient replacement for animals. However, little is known about the effect of administering *H. illucens* larvae on intestinal microbiota, bacterial metabolite profiles, and mucosal immune status in animals. This study aimed to investigate the effects of administering *H. illucens* larvae on colonic microbiota and bacterial metabolites production in finishing pigs. Seventy-two crossbred (Duroc × Landrace × Large White) female pigs (initial body weight, 76.0 ± 0.52 kg) were randomly allocated to three different dietary treatments: a control diet (Control group) and two diets corresponding to 4% (H1 group) and 8% (H2 group) *H. illucens* larvae inclusion levels, respectively. Each treatment consisted of eight pens (replicates), with three pigs per pen. After 46 days of feeding, eight pigs per treatment (*n* = 8) were slaughtered, and the colonic digesta and mucosa were collected for microbial composition and microbial fermentation products, and genes expression analyses.

**Results:**

The results showed that the H1 diet significantly increased the abundance of *Lactobacillus*, *Pseudobutyrivibrio*, *Roseburia*, and *Faecalibacterium* compared with those in the control group (*P* < 0.05), with a decrease in the abundance of *Streptococcus*. The numbers of *Lactobacillus*, *Roseburia*, and *Clostridium* cluster XIVa were significantly greater in the H1 group than in the control group (*P* < 0.05). Meanwhile, H2 diet increased the number of *Clostridium* cluster XIVa compared with the control group (*P* < 0.05). For colonic metabolites, total short chain fatty acids, butyrate, and isobutyrate concentrations were significantly higher in the H1 group than those in the control group (*P* < 0.05); the H1 treatment caused a striking decrease in protein fermentation compared with the control group, as the concentrations of total amines, cadaverine, tryptamine, phenol, *p*-cresol, and skatole were significantly lower (*P* < 0.05). Additionally, H2 diet also increased butyrate concentration compared with control group (*P* < 0.05), while decreased the concentrations of phenol, *p*-cresol, and skatole (*P* < 0.05). Pigs in the H1 group down-regulated the expression of *TLR*-4 and pro-inflammatory cytokines (*IFN-γ*) compared with pigs in the control group (*P* < 0.05), and up-regulated anti-inflammatory cytokine (*IL-10*) and intestinal barrier genes (*ZO-1*, *occludin*, and *mucin-1*). H2 diet up-regulated the expression of *ZO-1* compared with control group (*P* < 0.05). Furthermore, the changes in the colonic mucosal gene expression were associated with changes in the bacterial composition and their metabolites.

**Conclusions:**

Collectively, dietary inclusion of *Hermetia illucens* larvae may enhance mucosal immune homeostasis of pigs via altering bacterial composition and their metabolites. These findings provide a new perspective on insect meal as a sustainable protein source rich in nutrient ingredients for swine.

**Electronic supplementary material:**

The online version of this article (10.1186/s40104-019-0358-1) contains supplementary material, which is available to authorized users.

## Background

The global population will exceed 9 billion in 2050, so the demand for meat is expected to be 58% higher in 2050 than its 2010 level [1]. In parallel, this affects the demand for livestock feeds, and places heavy pressure on the already overexploited resources, especially protein sources. Therefore, more and more researchers are interested in finding new alternative and sustainable protein sources for livestock feed. Several insect species, such as the *Hermetia illucens*, as a sustainable food alternative and the feed ingredients are an interesting natural resource when considering their many environmental and health benefits [2, 3]. *H. illucens* larvae provide a high nutritive value feedstuff as they are rich in protein (40–44%) with a similar or even more balanced amino acid (AA) composition than soybean meal [4]. As the feed ingredients of a complete diet, *H. illucens* larvae have been found to modulate growth performance, nutrient digestibility, and blood profiles of chickens [5, 6]. Additionally, dietary including 4% and 8% *H. illucens* larvae in weaning piglets had no negative effects on the growth performance and nutrient digestibility [7]. Thus, these results demonstrate that *H. illucens* larvae can be used in the diet of monogastric animals as a potential valuable feed ingredient.

In addition, *H. illucens* larvae also contain bacteriostatic substances, such as chitin, which is a naturally occurring polysaccharide of the arthropod’s exoskeleton and considered to be one of the most abundant biopolymers in nature [8]. Chitin resists the digestion and absorption in the small intestine of monogastric animals, and enters the large intestine for use by the microbiota as a fermentable substrate. A previous study in piglets indicated that chitin derivatives (such as chitosan) administered can potentially reduce or inhibit the proliferation of pathogenic bacteria causing post weaning diarrhea [9]. Indeed, the effects of *H. illucens* larvae as a feed ingredient on the gut microbiota [5, 10, 11] and microbial metabolites (such as short-chain fatty acids, SCFAs) [5, 11] have been described in the cecal digesta of laying hens or broiler chickens, suggesting an evident effect of *H. illucens* larvae on the gut microbiota and metabolism. However, it remains unclear whether the *H. illucens larvae* meal as a dietary protein source has evident effect on the gut microbiota and its metabolism in the colon of finishing pigs.

As the first of innate immune response, the gastrointestinal epithelium represents a physical and an immunological barrier, and plays a significant role in maintenance and regulation of gastrointestinal health. Alteration of the bacterial community and their metabolite profile could affect intestinal immune function. Accumulating evidence has indicated that bacterial products resulting from carbohydrate fermentation, such as SCFAs exert many beneficial effects on physiological function of the intestine as they not only provide energy for epithelial cells, stimulate epithelial proliferation and tight junction formation, but also inhibit potential pathogen growth, inflammation and genotoxicity [12, 13], while bacterial metabolites from protein fermentation, such as amines, ammonia, indolic and phenolic compounds are considered to exert negative effects on the intestinal mucosa by interfering with its inflammatory responses [13, 14]. The previous study has indicated that *H. illucens* larvae as an alternative feed additive increased frequency of CD4+ lymphocyte in broiler chicks [15], which indicated that *H. illucens* larvae may have the potential to positively affect immune homeostasis. However, whether the *H. illucens larvae* as a dietary protein source would benefit mucosal immune responses in the gut remains limited and requires further investigation.

In the present study, we hypothesized that *H. illucens larvae* as a dietary protein source would change the bacterial community and its metabolites, and that these alterations can modulate the immune response in pigs. Therefore, the present study aimed to investigate the effects of *H. illucens larvae* as a dietary protein source on the microbiota, microbial fermentation profile, and mucosal immune responses in the colon of finishing pigs.

## Materials and methods

### Preparation of *H. illucens* larvae meal

*H. illucens* larvae were purchased from Guangzhou AnRuiJie Protection Technology Co., Ltd., (Guangzhou, Guangdong, China). The prepupae were dried at 80 °C for 30 min and air dried. Finally, the dried prepupae were crushed to powder through a 1.0-mm opening of a screen and was then used as *H. illucens* larvae meal, and then kept in a well-closed and light-resistant place. The chemical composition, energy content, and amino acid concentration of the *H. illucens* larvae are shown in Table 1.

**Table 1:** Analyzed chemical characteristics (% as fed), gross energy (MJ/kg), and amino acid concentration (% as fed) of the *Hermetia illucens* larvae meal used in the present study with finishing pigs

Items	*Hermetia illucens* larvae
Analyzed composition	
DM, %	92.22
Gross energy, MJ/kg	21.80
CP, %	34.97
Ether extract, %	35.49
Ash, %	6.46
ADF, %	6.71
ADF-linked protein, %	5.59
Chitin, %	4.65
Mineral composition, %	
Total P	0.83
Ca	4.39
Essential amino acids	
Lysine	2.10
Methionine	0.50
Methionine + Cystine	0.62
Isoleucine	1.36
Leucine	2.12
Tryptophan	0.60
Valine	1.86
Threonine	1.17
Arginine	2.30
Phenylalanine	1.18
Histidine	0.80
Non-essential amino acids	
Alanine	2.55
Aspartate	2.67
Glutamate	4.11
Glycine	1.61
Serine	1.16
Tyrosine	1.63

### Animals, diets, and sampling

A total of 72 crossbred (Duroc × Landrace × Large White) female pigs were randomly allocated to three different experimental diets based on their body weights (BW, 76.0 ± 0.52 kg). Pigs in the three treatments received increasing levels of *H. illucens* larvae meal (0, 4% and 8%; Control, H1, and H2 group, respectively). Each treatment consisted of eight pens (replicates), with three pigs per pen. All of the experimental diets were formulated to meet or exceed the nutrient recommendations of the National Research Council (Table 2) [17]. All pigs were provided the diet and water ad libitum during the 46-day experiment. The feed consumption per pen was recorded every day to calculate average daily feed intake (ADFI). The BWs of all pigs were recorded at the beginning and the end of the study period to determine average daily gain (ADG).

**Table 2:** Feed ingredient and nutrient composition of experimental diets (as-fed basis)

Items	Treatment^a^		
	Control	H1	H2
Ingredient, %			
Corn	71.00	71.20	71.76
Soybean meal	16.98	13.86	10.75
Wheat bran	6.00	6.00	6.00
*Hermetia illucens* L.	–	4.00	8.00
Soybean oil	2.50	1.70	0.50
Vitamin-mineral premix^b^	1.00	1.00	1.00
Dicalcium phosphate	0.95	0.95	1.00
Limestone	0.75	0.40	0.03
NaCl	0.30	0.30	0.30
*L*-Lysine HCl(98%)	0.38	0.40	0.42
*DL*-Methionine	0.06	0.07	0.09
*L*-Threonine	0.06	0.11	0.15
*L*-Tryptophan	0.02	0.01	–
Total	100.00	100.00	100.00
Calculated content^c^			
ME^d^, MJ/kg	14.37	14.38	14.38
Standard ileal digestible amino acid, %			
Lysine	0.73	0.73	0.73
Threonine	0.46	0.46	0.46
Methionine + Cysteine	0.42	0.42	0.42
Tryptophan	0.14	0.14	0.14
Analyzed nutrient composition^e^			
Dry matter, %	89.22	89.16	89.23
Crude protein, %	14.53	14.53	14.54
Ether extract, %	5.10	5.10	5.10
Crude ash, %	4.36	4.39	4.50
ADF, %	3.51	4.50	4.92
ADF-linked protein, %	0.85	1.58	2.89
Chitin, %	0.00	0.90	1.90

^a^diets H1 and H2 contained 4% and 8% *Hermetia illucens* larvae in an amount providing similar nitrogen to the diet as control diet, respectively

^b^Provided per kilogram of complete diet: vitamin A, 15,000 IU; vitamin D_3_, 3,000 IU; vitamin E, 150 mg; vitamin K_3_, 3 mg; vitamin B_1_, 3 mg; vitamin B_2_, 6 mg; vitamin B_6_, 5 mg; vitamin B_12_, 0.03 mg; niacin, 45 mg; vitamin C, 250 mg; calcium pantothenate, 9 mg; folic acid, 1 mg; biotin, 0.3 mg; choline chloride, 500 mg; Fe (FeSO_4_.H_2_O), 170 mg; Cu (CuSO_4_.5H_2_O), 150 mg; I (KI), 0.90 mg; Se (Na_2_SeO_3_),0.2 mg; Zn (ZnSO_4_.H_2_O), 150 mg; Mg (MgO), 68 mg; Mn (MnSO_4_.H_2_O), 80 mg; Co (CoCl_2_), 0.3 mg

^c^Values were based on the chemical analysis

^d^ME = metabolized energy

^e^Analytical results obtained according to AOAC [16]

At the end of the feeding period (day 46), one pig from each pen was randomly selected and euthanized by electrical stunning and exsanguination after fasting approximately 12 h. The digesta of the colon were collected, homogenized, and stored at − 80 °C for later determination of the bacterial communities using Illumina MiSeq sequencing and microbial metabolites analyses. A sterile glass microscope slide was used to scrape mucosa as previously described [18], immediately frozen in liquid nitrogen for later gene expression.

### Chemical composition analysis

The common nutritional components (dry matter, DM; crude protein, CP; crude fat; ash; and acid detergent fiber, ADF) in the experimental diets and *H. illucens* larvae were analyzed according to the Association of Official Analytical Chemists (AOAC) procedures [16]. To determine the amino acid concentrations in *H. illucens* larvae meal (except methionine and tryptophan), approximately 1.0 g of each sample was digested in 10 mL of 6 mol/L HCl at 110 °C for 24 h; the methionine concentration was measured after oxidation with performic acid, and tryptophan concentrations were determined after alkaline hydrolysis according to the AOAC [16]. The protein linked to ADF in the experimental diets and *H. illucens* larvae were determined [19], and used to estimate the amount of chitin according to previous study [20].

Colonic SCFAs concentrations were determined by gas chromatography (GC) according to a previous study [21]. Lactate concentration in the colonic digesta was measured using a commercial assay kit (Nanjing Jiancheng Biological Engineering Institute, Nanjing, China) according to the manufacturer’s instructions. Ammonia concentration in the colonic digesta was measured using UV spectrophotometer according to a previous method [22]. The biogenic amines concentrations in the colonic digesta were analyzed using high-performance liquid chromatography (HPLC) with precolumn dansylation according to a previous study [23]. The concentrations of phenolic and indolic compounds were analyzed by HPLC, as described previously [24], with slight modifications. Briefly, 0.1 g of the colonic digesta was mixed with 1.0 mL acetonitrile. The mixture was vortexed and stored at − 20 °C for 20 min and then centrifuged at 3,000×*g* for 10 min at 4 °C. The supernatant was filtered through a 0.22-μm membrane and then analyzed on a Waters Alliance system (Alliance HPLC System e2695 separation module; Waters, Milford, MA, USA) with a multi λ fluorescence detector (Waters 2475). Gradient elution of two mobile phases was used: mobile phase A consisted of HPLC grade water, and mobile phase B was acetonitrile. The gradient program was: 82% A initially, 55% A at 12 min, 10% A at 22 min, and 100% B at 23 min. The flow rate was 1.0 mL/min, and column temperature was 30 °C.

### DNA extraction, Illumina MiSeq sequencing, and data processing

Total genomic DNA in the colonic digesta was extracted using the QIAamp PowerFecal DNA Kit (QIAGEN, Hilden, Germany), according to the manufacturer’s instructions. DNA concentrations of all samples were quantified with the Nanodrop 1000 spectrophotometer (Thermo Fisher Scientific Inc., Wilmington, DE, USA). The V3-V4 regions of all bacterial 16S rRNA genes were amplified by polymerase chain reaction (PCR) using the 338F universal forward primer (5′-ACTCCTRCGGGAGGCAGCAG-3′) and the 806R universal reverse primer (5′-GGACTACCVGGGTATCTAAT-3′) [25]. The PCR products were purified using the AxyPrep DNA Gel Extraction Kit (Axygen Biosciences, Union City, CA, USA), according to the manufacturer’s instructions. The purified amplicons were pooled in equimolar concentrations from each sample and paired-end sequenced (2 × 250) on an Illumina MiSeq platform (Majorbio, Shanghai, China) according to standard protocols [26].

The raw sequence data generated from 16S rRNA MiSeq sequencing were demultiplexed and quality-filtered using the QIIME (version 1.17) software package [27]. Gaps in each sequence were discarded from all samples to decrease noise by screening, filtering, and pre-clustering processes as described by a previous study [28]. Operational taxonomic units (OTUs) were clustered with a cut-off of value 97% similarity using UPARSE (version 7.1, http://drive5.com/uparse/), and chimeric sequences were identified and removed using UCHIME [29]. The representative sequence of each OTU was analyzed with the Ribosomal Database Project Classifier (RRID: SCR_006633) against the Silva (SSU119) 16S rRNA database at a confidence level of 90%.

Bacterial diversity, including a rarefaction analysis, richness estimators (Chao 1 and ACE), and diversity indices (Shannon and Simpson) were calculated using MOTHUR software (version 1.35.1, http://www.mothur.org), according to previous instructions [30]. The principal coordinate analysis (PCoA) was performed based on the Bray−Curtis distance, and analysis of molecular variance (AMOVA) was performed to compare the dissimilarities among the specimens [30].

The 16S sequencing data in this study were deposited in the National Center of Biotechnology Information (NCBI) Sequence Read Archive (SRA) database under accession number PRJNA504488.

### Quantitative real-time PCR

The quantities of total bacteria, Firmicutes, *Ruminococcus, Bacteroides-Prevotella*, *Clostridium* cluster IV, *Clostridium* cluster XIVa, *Bifidobacterium*, *Escherichia coli* and *Lactobacillus* from each sample of colonic digesta (*n* = 8) were quantified by real-time quantitative PCR (qPCR) using specific primers (Additional file 1: Table S1). qPCR was performed on the CFX96 Real-Time PCR Detection System (Bio-Rad, Hercules, CA, USA) with TB Green™ Premix Ex Taq™ (Takara Biotechnology, Dalian, China). The reaction mixtures and qPCR conditions were set according to a previous study [21].

### Mucosal RNA extraction of colon and real-time PCR

Total RNA was extracted from the colonic mucosa with the TRIzol reagent (Takara Biotechnology, Dalian, China) according to the manufacturer’s instructions. The RNA quality of every sample was quantified by Nanodrop 1000 spectrophotometer (Thermo Fisher Scientific Inc., Wiington, DE, USA), and the ratio (OD_260_: OD_280_) ranged from 1.8 to 2.0. Thereafter, 1 μg total RNA was reverse-transcribed to cDNA using a Synthesis Kit (Takara Biotechnology, Dalian, China) according to the manufacturer’s instructions. Primers used for selected genes were presented in Additional file 2: Table S2 in the supplemental material. Real-time PCR of the target genes and β-actin was performed on CFX96 Real-Time PCR Detection System (Bio-Rad, Hercules, CA, USA) with TB Green™ Premix Ex Taq™ (Takara Biotechnology, Dalian, China). The reaction mixtures and real-time PCR conditions were set according to a previous study [18]. The housekeeping gene β-actin was used to normalize the expression levels of each target gene, and according to the following formula 2^−(∆∆Ct)^, where ∆∆Ct = (Ct_target_– Ct_β-actin_)_treatment_ – (Ct_target_– Ct_β-actin_)_control_.

### Data analysis

All experimental data were analyzed using the SPSS software package (SPSS v. 20.0, SPSS Inc., Chicago IL, USA). The normality of the data distribution was confirmed by the Shapiro−Wilk test before assessing differences between the groups. The Kruskal−Wallis One-way analysis of variance (ANOVA) with the Benjamini−Hochberg false discovery rate (FDR) multiple-testing correction [31] was used to analyze non-normally distributed variables (some taxa richness data). Bacterial abundance data (at the phylum, genus, and species levels) are expressed as medians; a *P-*value < 0.05 was regarded as significant.

The bacterial gene copy, metabolite concentration (lactate, SCFAs, ammonia, amines, and phenol and indole compounds), and gene expression data that were normally distributed were analyzed by One-way ANOVA with a Tukey’s *post hoc* test. Data are expressed as means ± standard error of mean (SEM), and *P* ≤ 0.05 was regarded as significant. Correlations between mucosal gene expression and significantly changed bacterial abundances (at the genus level and qPCR, *q* < 0.05) and fermentation end-products by dietary treatment were analyzed by Spearman’s correlation analysis using GraphPad Prim version 5.0 (GraphPad Software, San Diego, CA, USA). A correlation was considered significant when the absolute Spearman’s correlation coefficient was > 0.5 and significant at *P* < 0.05.

## Results

### Growth performance

During the whole trial, the pigs were considered healthy and no mortality has been observed. In this study, the H1 diet significantly increased (*P* < 0.05) the ADG of pigs (means ± SEM: 0.89 ± 0.03 kg/d, 0.98 ± 0.03 kg/d, 0.86 ± 0.02 kg/d in control, H1, and H2 group, respectively) and decreased (*P* < 0.05) the feed conversion rate (F:G) (3.21 ± 0.06, 2.85 ± 0.12, 3.23 ± 0.05 in control, H1, and H2 group, respectively) compared with control and H2 diets. However, no difference in the ADFI (One-way ANOVA, *P* = 0.466) was observed between the control group (means ± SEM: 2.83 ± 0.05 kg/d), H1 group (means ± SEM: 2.77 ± 0.03 kg/d), and H2 group (means ± SEM: 2.87 ± 0.04 kg/d).

### Microbiota composition of the colonic digesta

**Fig. 1:**
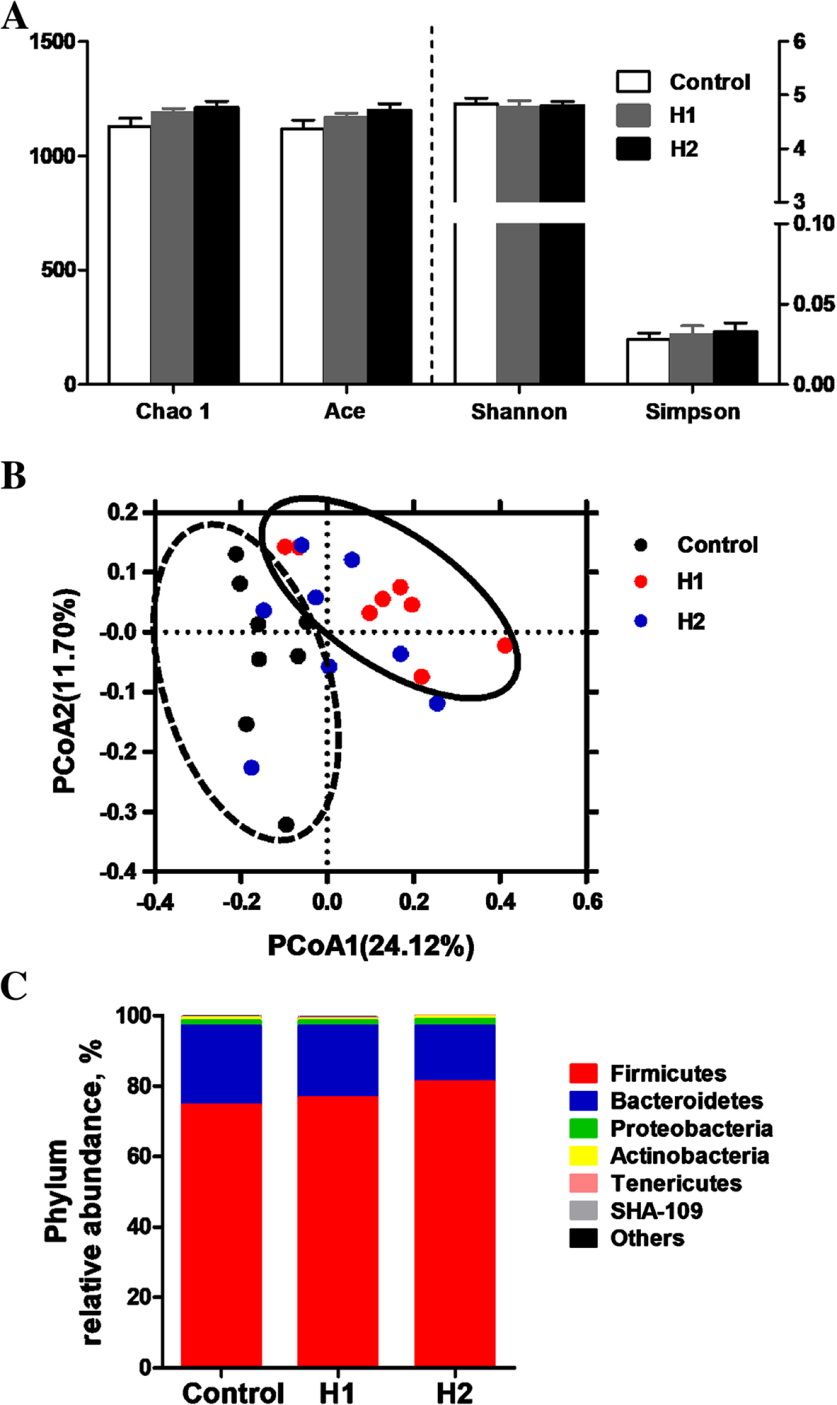
**a** Diversity and richness of colonic digesta microbial community at the 3% dissimilarity level. The values are means ± SEM (*n* = 8). **b** Principal coordinates analysis (PCoA) of bacterial communities in the colonic digesta of pigs (based on the Bray-Curtis distance). Circles with dash line or solid indicate that groups were significantly distinct using AMOVA analysis (*P* < 0.05). **c** Phylum-level relative abundance of 16S rRNA gene sequences from the colonoc digesta of pigs

The microbial composition of the colonic digesta following the *H. illucens* larvae treatment was revealed by 16S rRNA Illumina MiSeq sequencing. In the present study, a total of 1,031,156 V3-V4 16S rRNA effective sequences from the 24 samples, with an average 42,954 sequences per sample, were used for subsequent analysis. The flattened rarefaction curves showed that the sampling in each group provided sufficient OTU coverage (Additional file 3: Figure S1). No significant differences in species richness (as reflected by the ACE and Chao1 indices) or diversity (as reflected by Shannon and Simpson indices) were observed in the colonic digesta bacteria at the taxonomic level (Fig. 1a). However, the PCoA with the Bray−Curtis distance results showed that the H1 group was separated from the control group (Fig. 1b). AMOVA analysis also showed significant dissimilarities between the H1 and control groups (*F*s = 2.25, *P* < 0.01, among control, H1, and H2 groups; *F*s = 1.63, *P* < 0.05, H1 vs. H2; *F*s = 3.45, *P* < 0.001, control vs. H1; *F*s = 1.61, *P* = 0.069, control vs. H2).

At the phylum level, the Firmicutes and Bacteroidetes were the two dominant phyla, contributing 74.99% and 22.09% to the control group, 75.75% and 21.40% to the H1 group, and 78.54% and 17.93% to the H2 group, respectively (Fig. 1c). Proteobacteria and Actinobacteria were the next two most dominant phyla, accounting for 1.78% and 0.19% in the control group, 1.69% and 0.55% in the H1 group, 2.17% and 0.66% in the H2 group, respectively. No significant changes were found in the abundances of any of the phyla.

**Fig. 2:**
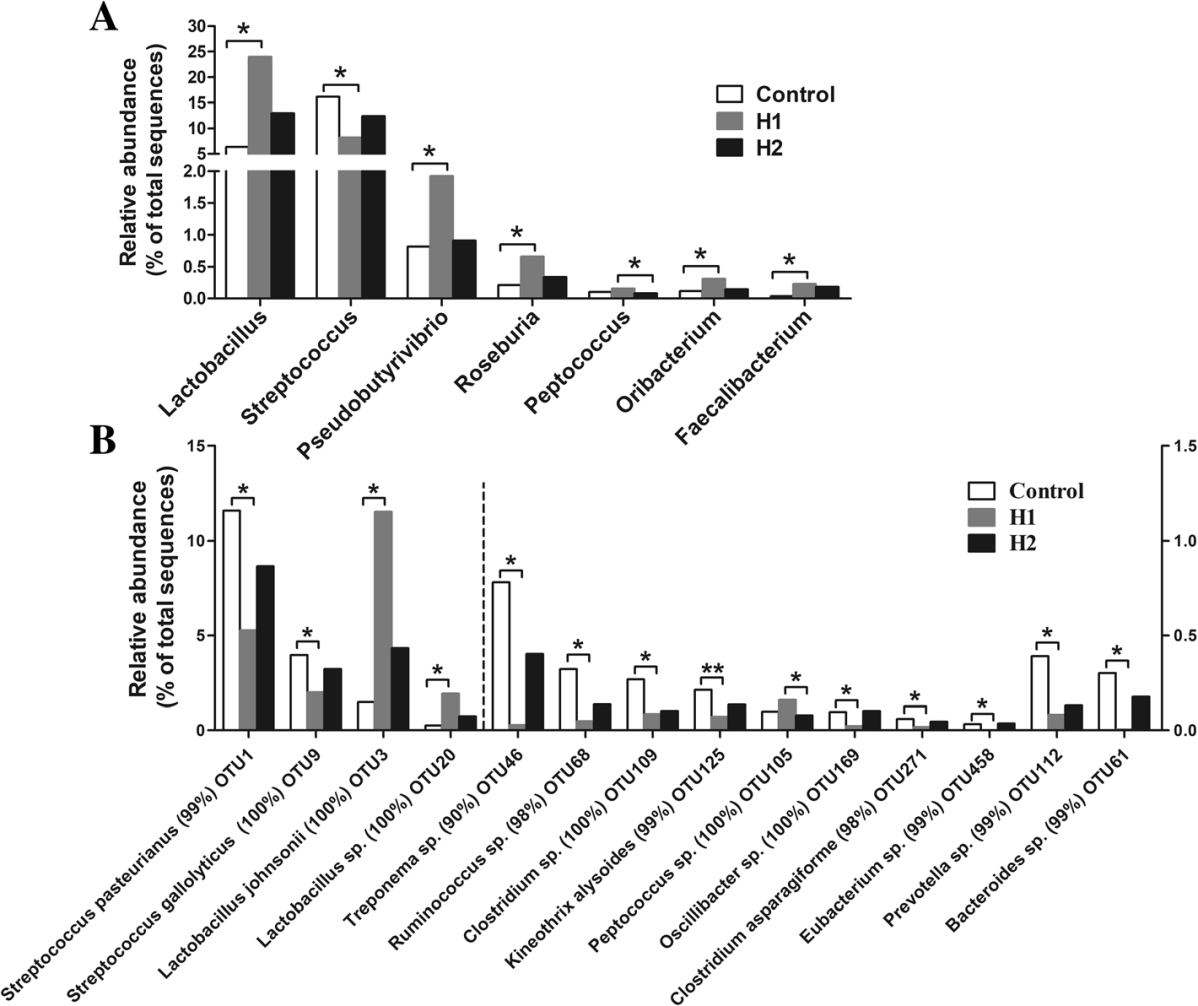
Significantly changed bacteria genera (**a**) and species (**b**) by *Hermetia illucens* larvae treatments. The values were expressed as the medians (*n* = 8). Asterisks indicated statistical differences between different group (Kruskal-Wallis test): *FDR-adjusted *P*-value < 0.05; ** FDR-adjusted *P*-value < 0.01. OTU, operational taxonomic unit

At the genus level, the 30 most predominant genera in the colonic digesta are presented as a heat map (Additional file 4: Figure S2). The 10 most predominant genera (those with a relative abundance ≥3% in at least one treatment) were *Lactobacillus*, *Streptococcus*, unclassified Ruminococcaceae, unclassified Lachnospiraceae, *Clostridium*_sensu_stricto_1, unclassified Prevotellaceae, unclassified S24–7, *Prevotella*, unclassified Peptostreptococcaceae, and unclassified Christensenellaceae. The relative abundance of *Lactobacillus*, *Pseudobutyrivibrio*, *Roseburia*, *Oribacterium*, and *Faecalibacterium* increased significantly in pigs fed the H1 diet (*P* < 0.05) compared with control diet, but the abundance of *Streptococcus* decreased (*P* < 0.05) (Fig. 2a). Meanwhile, the H1 diet resulted in a higher abundance of *Peptococcus* than that of the H2 diet (*P* < 0.05).

At the species level, a total of 1505 OTUs was identified in the colonic digesta. As shown in Fig. 2b, the relative abundance of OTUs closely related to *Streptococcus pasteurianus*, *Streptococcus gallolyticus*, *Treponema* sp., *Ruminococcus* sp., *Clostridium* sp., *Kineothrix alysoides*, *Oscillibacter* sp., *Clostridium asparagiforme*, *Eubacterium* sp., *Prevotella* sp., and *Barnesiella* sp. (Fig. 2b) significantly decreased in pigs fed the H1 diet (*P* < 0.05) compared with the control diet, while the relative abundance of OTUs closely related to *Lactobacillus johnsonii* and *Lactobacillus* sp. increased (*P* < 0.05). Meanwhile, the H1 diet also increased (*P* < 0.05) the abundance of OTU closely related to *Peptococcus* sp. compared with the H2 diet.

**Fig. 3:**
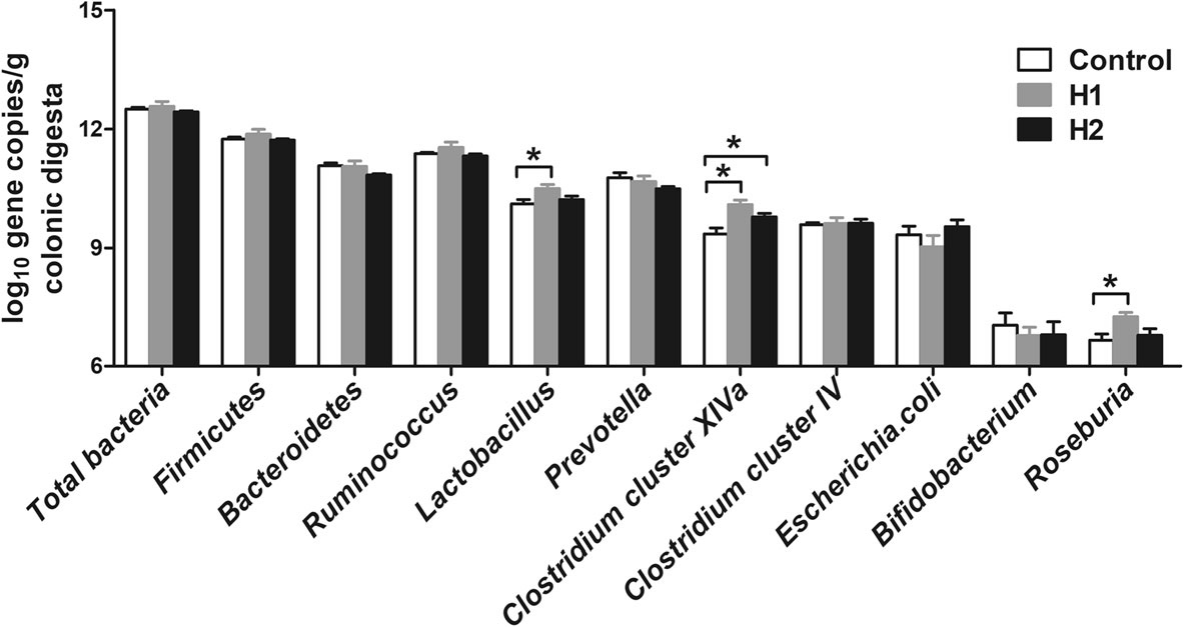
Response of the copy numbers (log_10_ gene copies/g digesta sample) of major bacterial taxonomic groups in the colonic digesta of pigs toward *Hermetia illucens* larvae treatments. The values are means ± SEM (*n* = 8). Asterisks indicated statistical differences between different group (One-way ANOVA with a Tukey *post hoc* test): *P < 0.05

Some key bacteria groups were determined by qPCR to identify the quantitative changes in the bacterial groups in the colonic digesta following treatment with *H. illucens* larvae. As shown in Fig. 3, the H1 diet significantly increased (*P* < 0.05) the numbers of *Lactobacillus*, *Clostridium* cluster IV, and *Roseburia* compared with the control diet. The H2 diet also increased (*P* < 0.05) the number of *Clostridium* cluster IV compared with the control diet. However, no significant differences (*P* > 0.05) were observed in the numbers of total bacteria, Firmicutes, Bacteroidetes, *Ruminococcus*, *Prevotella*, *Clostridium* cluster XIVa, *E. coli*, or *Bifidobacterium* among the different dietary treatments. Taken together, these results indicate that a diet including 4% *H. illucens* larvae meal significantly changed the colonic digesta bacterial community structure.

### Fermentation metabolites in the colonic digesta

**Fig. 4:**
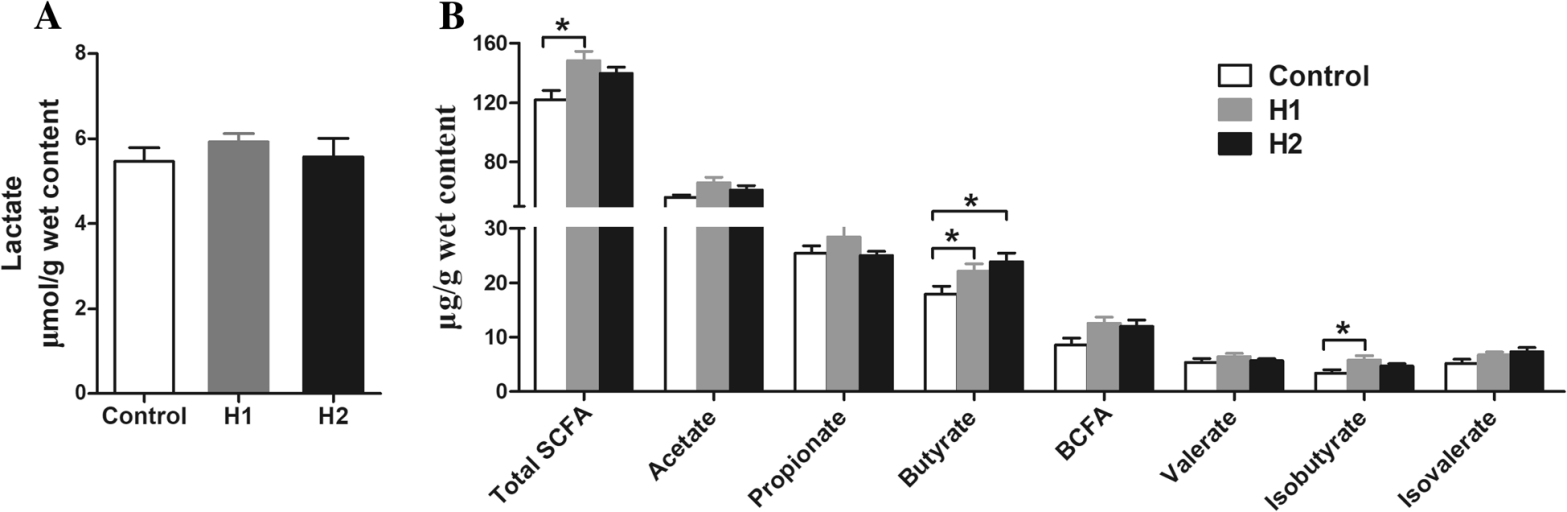
Effect of the dietary inclusion of *Hermetia illucens* larvae on the concentration of lactate (**a**) and short-chain fatty acid (SCFA) (**b**) in the colonic digesta of pigs. The values are means ± SEM (*n* = 8). Asterisks indicated statistical differences between different group (One-way ANOVA with a Tukey *post hoc* test): *P < 0.05. Abbreviations: Total SCFA, total short-chain fatty acid; BCFA, branched-chain fatty acid

To explore whether feeding *H. illucens* larvae affects the fermentation profile of the colonic digesta, some microbial metabolites were determined. For SCFAs (Fig. 4b), the concentrations of total SCFAs, butyrate, and isobutyrate increased significantly in pigs fed the H1 diet (*P* < 0.05) compared with the control diet. The concentration of butyrate also increased (*P* < 0.05) in response to the H2 diet compared with the control diet. However, the dietary treatment did not affect the acetate, propionate, branch-chain fatty acid (BCFA), valerate, or isovalerate concentrations (*P* > 0.05). The concentration of lactate in the colonic digesta was not affected by the dietary treatments (Fig. 4a).

**Fig. 5:**
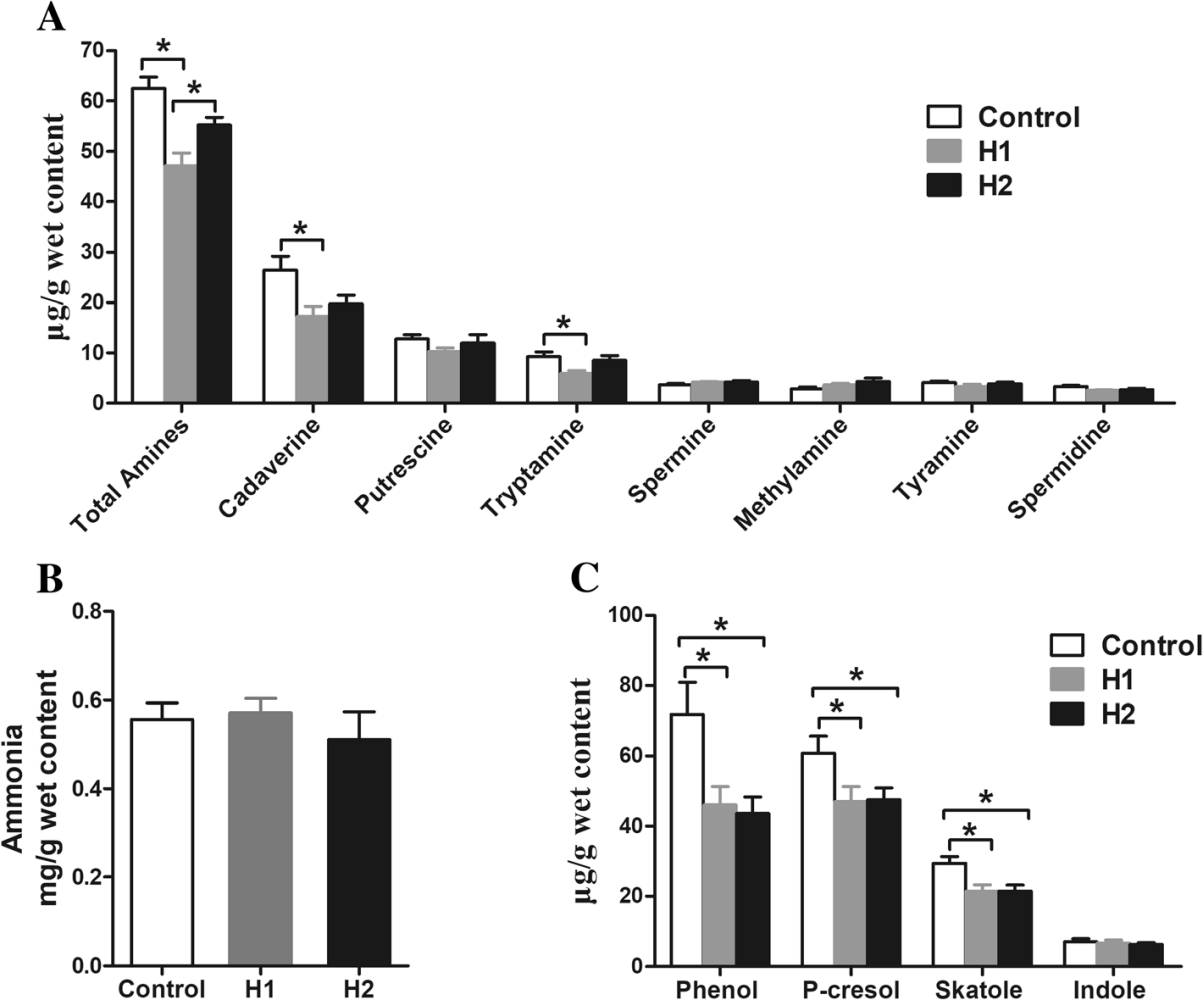
Effect of the dietary inclusion of *Hermetia illucens* larvae on the concentration of amino acid fermentation products in the colonic digesta of pigs: biogenic amines (**a**), ammonia (**b**), and phenolic and indole compounds (**c**). The values are means ± SEM (*n* = 8). Asterisks indicated statistical differences between different group (One-way ANOVA with a Tukey *post hoc* test): *P < 0.05

Biogenic amines and ammonia are produced from bacterial decarboxylation and deamination of amino acids, respectively. For biogenic amines (Fig. 5a), cadaverine, putrescine, and tryptamine were the major biogenic amines in the colonic digesta. The concentrations of total amines, cadaverine, and tryptamine decreased significantly (*P* < 0.05) in pigs fed the H1 diet compared with the control diet, as well as the concentration of total amines decreased (*P* < 0.05) compared with the H2 diet. However, the concentrations of putrescine, spermine, methylamine, tyramine, and spermidine were not affected by the different dietary treatments (*P* > 0.05). The dietary treatments also did not affect the ammonia concentration (Fig. 5b).

Phenol and indole compounds are derived from metabolism of aromatic amino acids by intestinal bacteria. As shown in Fig. 5c, the H1 and H2 diets significantly decreased (*P* < 0.05) the concentrations of phenol, *p*-cresol, and skatole compared with the control diet. However, the concentration of indole was not affected by the different dietary treatments (*P* > 0.05). Overall, these results indicate that a diet including *H. illucens* larvae markedly increased the concentrations of SCFAs (total SCFAs, butyrate, and isobutyrate), but decreased the concentrations of biogenic amines as well as phenol and indole compounds in the colonic digesta, suggesting a strong impact of *H. illucens* larvae on carbohydrate and amino acid metabolism characteristics in the colon.

### Gene expression in colonic mucosa

**Fig. 6:**
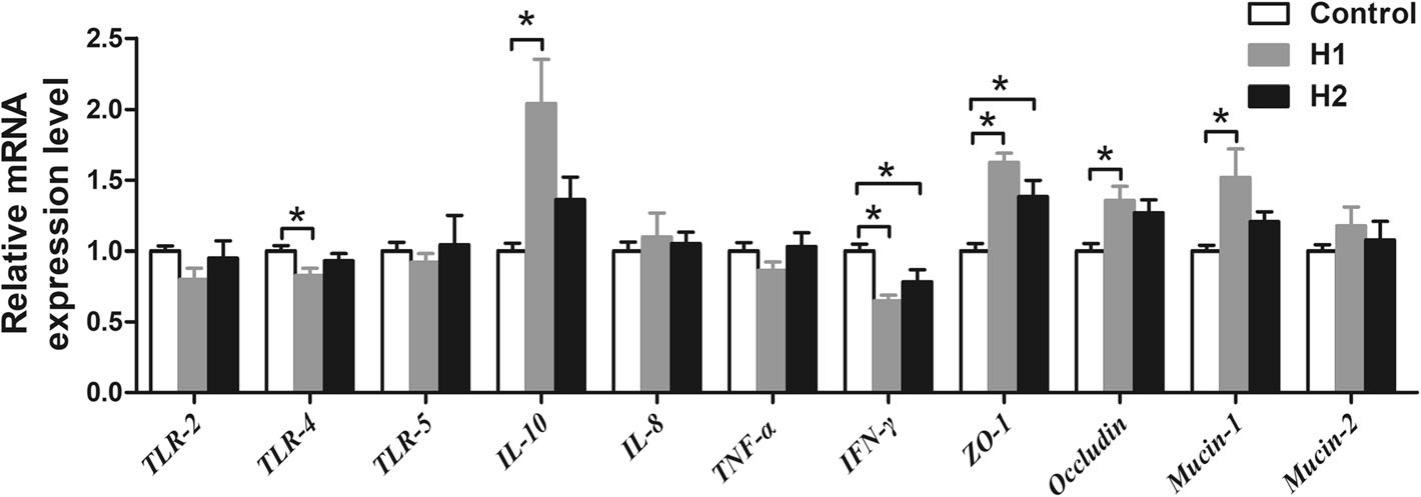
Effect of the dietary inclusion of *Hermetia illucens* larvae on the relative mRNA expression of genes related to TLR, cytokines, and barrier function in the colonic mucosa. Asterisks indicated statistical differences between different group (One-way ANOVA with a Tukey *post hoc* test): *P < 0.05

To assess whether feeding *H. illucens* larvae affects the intestinal immune function, the mRNA expression of several TLR, cytokines, and tight junction proteins were determined. As shown in Fig. 6, the H1 diet significantly down-regulated the relative mRNA expression of *TLR-4* and *IFN-γ* compared with control diet (*P* < 0.05), while up-regulated the relative mRNA expression of *IL-10*, *ZO-1*, *occludin*, and *mucin-1* (*P* < 0.05). The H2 diet also down-regulated the relative mRNA expression of *IFN-γ* compared with control diet (*P* < 0.05), while up-regulated the relative mRNA expression *ZO-1* (*P* < 0.05). However, there was no significant difference in the expression of *TLR-2*, *TLR-5*, *IL-8*, *TNF-α*, and *mucin-2* among the three groups (*P* > 0.05).

### Correlation analysis between mucosal gene expression with colonic microbiome or bacterial fermentation metabolites

**Fig. 7:**
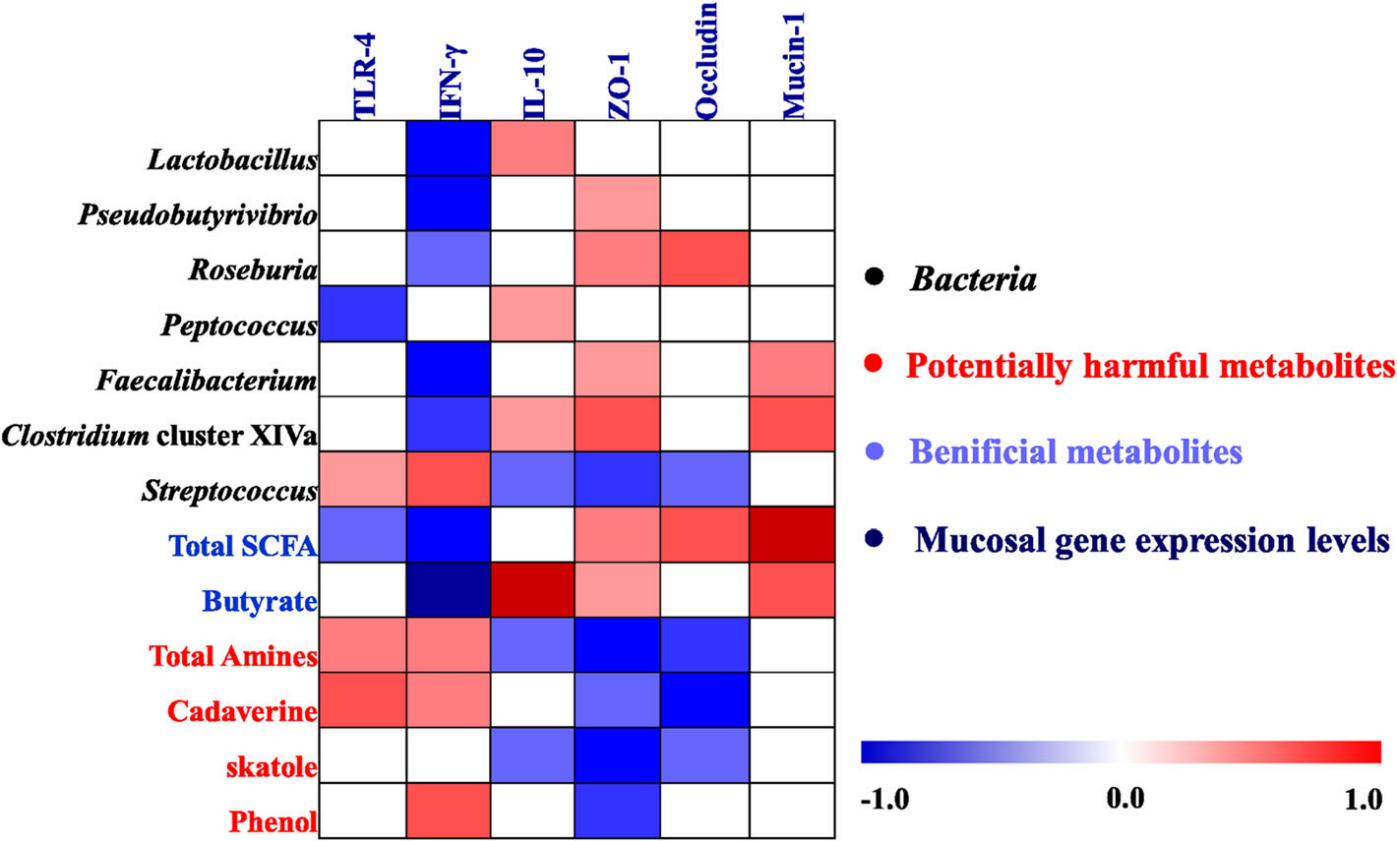
Spearman’s correlation analysis between the abundance of colonic microbiota (at the genus level and qPCR), microbial metabolites and mucosal gene expression level affected by dietary treatment. Cells are colored based on the correlation coefficient between the significantly changed bacteria (the relative abundance and the numbers of bacteria) and metabolites (concentrations) and mucosal gene expression level. The intensity of the colors represents the degree of association. Red represented a significant positive correlation (*P* < 0.05), blue represents significantly negative correlation (*P* < 0.05), and white shows that the correlation was not significant (*P* > 0.05). Total SCFA: total short-chain fatty acids

A Spearman’s rank correlation analysis matrix was carried out to determine whether there was any relationship among mucosal gene expression and microbial composition (abundance of bacterial genera and qPCR results) and the concentrations of metabolites (Fig. 7). The correlation analysis revealed that the mRNA expression level of *TLR-4* was positively correlated with *Streptococcus*, total amines, cadaverine, skatole, and phenol (*P* < 0.05), while negatively correlated with *Peptococcus* and total SCFA (*P* < 0.05). The mRNA expression level of *IFN-γ* was positively correlated with *Streptococcus*, total amines, cadaverine, and phenol (*P* < 0.05), while negatively correlated with *Lactobacillus*, *Pseudobutyrivibrio*, *Roseburia*, *Faecalibacterium*, *Clostridium* cluster XIVa, total SCFA, and butyrate (*P* < 0.05). The mRNA expression level of *IL-10* was positively correlated with *Lactobacillus*, *Peptococcus*, *Clostridium* cluster XIVa, and butyrate (*P* < 0.05), while negatively correlated with *Streptococcus*, total amines, cadaverine, and phenol (*P* < 0.05). Furthermore, the mRNA expression level of *ZO-1* showed positive correlations with *Pseudobutyrivibrio*, *Roseburia*, *Faecalibacterium*, *Clostridium* cluster XIVa, total SCFA, and butyrate (*P* < 0.05), while showed negative correlations with *Streptococcus*, total amines, cadaverine, skatole, and phenol (*P* < 0.05). The mRNA expression level of *occludin* was positively correlated with *Roseburia* and total SCFA (*P* < 0.05), while negatively correlated *Streptococcus*, total amines, cadaverine, and skatole (*P* < 0.05). The mRNA expression of *mucin-1* was positively correlated with *Faecalibacterium*, *Clostridium* cluster XIVa, total SCFA, and butyrate (*P* < 0.05). Additionally, the correlation between bacteria and the concentrations of metabolites were also analyzed and showed in supplementary Additional file 5: Figure S3. Collectively, these results indicate that the changes in the colonic digesta microbiota and metabolites were correlated with alterations of epithelial gene expression in pig.

## Discussion

**Fig. 8:**
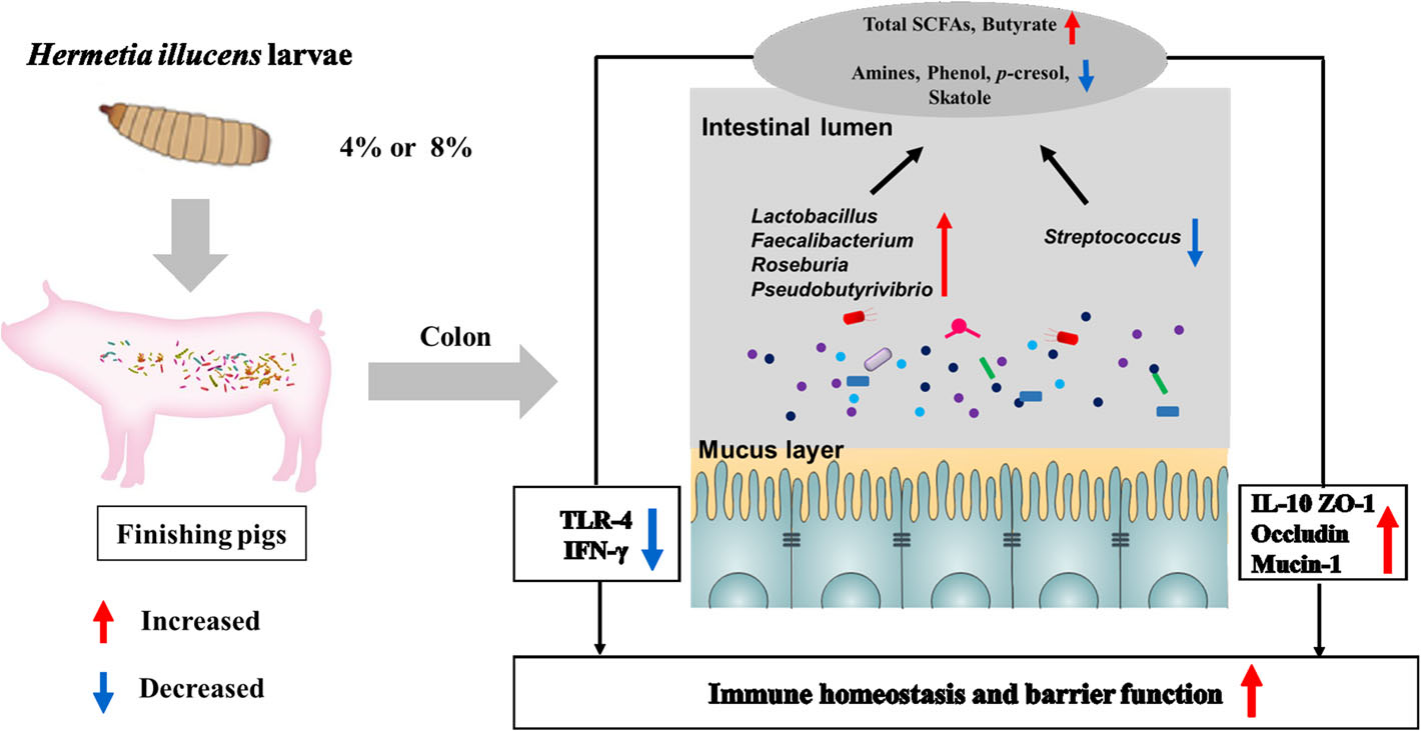
Proposed function model of *Hermetia illucens* larvae meal. Items with blue down-arrow indicated the decreased bacteria, metabolites, or genes expression in the *Hermetia illucens* larvae diet group compared with the control diet, whereas those with a red up-arrow indicated the decreased ones in the *Hermetia illucens* larvae diet group. Total SCFAs: total short-chain fatty acids; *IFN-γ*: interferon-γ; *IL-10*: interleukin-10; *TLR-4*: Toll-like receptors-4; *TNF-α*, tumor necrosis factor-α; *ZO-1*: Zona occludens protein 1

*H. illucens larvae* are a novel source of dietary protein that have been used widely in the diets of animals, such as broiler chickens, laying hens, and weaning piglets [2]. The present study, used 16S rRNA Miseq sequencing and biochemical analyses, for the first time to identify the changes in the microbiota and metabolites of the colonic digesta and mucosal immune genes expression in finishing pigs in response to administration of *H. illucens* larvae. Our results showed that dietary supplementation with 4% *H. illucens* larvae dramatically increased the abundance and numbers of *Lactobacillus* and some butyrate-producing bacteria (*Pseudobutyrivibrio*, *Roseburia*, and *Faecalibacterium*), SCFAs concentrations, anti-inflammatory cytokine, and intestinal barrier genes expression in the colon, but decreased the abundance of *Streptococcus*, the concentrations of nitrogenous fermentation products (cadaverine, tryptamine, phenol, *p*-cresol, and skatole), and pro-inflammatory cytokine genes expression. The changes in the colonic mucosal gene expression were associated with changes in bacterial composition and their metabolites (Fig. 8). These findings suggest that feeding low levels (about 4%) of *H. illucens* larvae in the diet may act as a potential prebiotic for finishing pigs.

### *H. illucens* larvae significantly alter the colonic microbiota structure of finishing pigs

Diet composition is the major factor that affects the gastrointestinal tract microbiota [32]. In the present study, a diet including 4% *H. illucens* larvae caused a significant difference in the colonic microbial composition compared with the control group, as revealed by the Bray–Curtis PCoA and AMOVA analyses. The univariate statistical analysis indicated that a *H. illucens* larvae meal (in 4% supplementation) markedly increased the abundance of many potentially beneficial bacteria and reduced the abundance of potentially opportunistic pathogens compared with control diet. Among the genera affected by the *H. illucens* larvae, bacterial species, such as *Lactobacillus* markedly increased in the H1 pigs (Fig. 2a). The *H. illucens* larvae treatment also increased the numbers of *Lactobacillus* in laying hens by an average of 7.44-fold [10]. *Lactobacillus* mainly exerts health-promoting effects on gut physiology and is generally considered a potentially beneficial microbe. Thus, these results suggest that *H. illucens* larvae increased the potentially beneficial bacteria. Meanwhile, *H. illucens* larvae also increased some butyrate-producing bacteria, such as *Roseburia*, *Pseudobutyrivibrio*, and *Faecalibacterium* in the colon (Fig. 2a). Some species within the genera *Roseburia*, *Pseudobutyrivibrio*, and *Faecalibacterium* together with butyrate, are essential for maintaining the intestinal metabolism [33], and have many beneficial effects for colonic homeostasis by promoting epithelial energy metabolism and stimulating immune development [34]. In addition, another important finding was that *H. illucens* larvae significantly increased the number of *Clostridium* cluster XIVa bacteria (Fig. 3). Bacteria within *Clostridium* cluster XIVa are regarded as a predominant population and are butyrate producers [35]. which play a crucial role in maintaining gut health. Therefore, the increase in the abundance of *Roseburia*, *Pseudobutyrivibrio*, and *Faecalibacterium* and the number of *Clostridium* cluster XIVa in the colon indicate that carbohydrate metabolism by bacteria may have been affected by administration *H. illucens* larvae.

On the other hand, diet including 4% *H. illucens* larvae markedly decreased the abundance of *Streptococcus* compared with the control diet (Fig. 2a). Some bacteria within the genus S*treptococcus*, including *S. gallolyticus* [36] and *S. pasteurianus* [37] are opportunistic pathogens that induce diarrhea in pigs or are associated with digestive tract malignancies, respectively. An increase in the abundance of S*treptococcus* has been reported in the feces of patients with colorectal cancer [38]. Thus, the decrease in the abundance of *Streptococcus* suggests that the dietary inclusion of 4% *H. illucens* larvae may have beneficial to intestinal health for finishing pigs.

Until now, the exact mechanism for the change in the colonic microbiota by *H. illucens* larvae has remained unclear. *H. illucens* larvae contain chitin (about 4.65% in the present study), which serves as a substrate for gut microbiota and then alters their composition. Previous studies have indicated that low-level chitin restores the compositional balance in the microbial communities of humans [39] or mice [40]. *H. illucens* larvae also have a high concentration of the medium-chain fatty acid (MCFA) lauric acid (C12:0, about 28% of total fatty acids in the present study, unpublished data), which is a natural antimicrobial agent and particularly active against Gram-positive bacteria [41, 42]. Furthermore, *H. illucens* larvae are also a potential source of antimicrobial peptides (AMP) and have broad-spectrum activity against both Gram-positive and Gram-negative bacteria [43]. Therefore, administering *H. illucens* larvae regulates the microbiota in the colon probably through chitin, lauric acid, and/or AMPs, but should be further studied to clarify the underlying mechanisms.

Additionally, we found that dietary inclusion of 8% *H. illucens* did not result in beneficial effects on the colonic microbiota in the current trial. To the best of our knowledge, the current study is the first to test partially *H. illucens* larvae inclusion in the diets of finishing pigs. Therefore, the available information on the effect of dietary *H. illucens* larvae inclusion on gut microbiota of finishing pigs is very limited. As mentioned above, *H. illucens* larvae contain chitin, which is not digestible by monogastric animals. A previous study has indicated that low concentrations of chitin might increase the number of *Lactobacillus* and decrease *E.coli* and *Salmonella* in the intestine of broilers, while high concentrations of chitin have adverse effects on these bacteria [44]. In weaning piglets, 8% *H. illucens* larvae diet had lower counts of Lactobacilli than the control and 4% *H. illucens* larvae diet [7]. Thus, dietary inclusion of 8% *H. illucens* larvae had no positive effects on the microbiota may be due to the higher level of chitin in the diets. Additionally, recent studies have indicated that insects contain non-protein nitrogen, such as chitin, nucleic acids, ammonia, nitrites, etc., could lead to an overestimation of the insect protein content [45, 46]. In the present study, we used the conventional nitrogen-to-protein conversion factor value of 6.25 to express the protein content of *H. illucens* larvae, which may result the true protein content in the diet and colonic digesta were lower in the H2 group than in the control group. A previous study has showed that non-protein nitrogen can inhibit the growth of beneficial bacteria, such as *Lactobacillus*, *Bifidobacterium*, and *Megasphaera* [47] Therefore, additionally potential explanation for had no beneficial effects on the colonic microbiota may be an increasing of non-protein nitrogen content when pigs fed the dietary inclusion of 8% *H. illucens* larvae, but further study will be required to elucidate the underlying mechanisms. Nevertheless, our findings suggest that dietary inclusion of 4% *H. illucens* larvae altered the microbiota structure of finishing pigs toward a host-friendly gut environment.

### *H. illucens* larvae significantly increase SCFA concentrations in the colon of finishing pigs

A change in intestinal microbiota is often accompanied by changes in microbial metabolism [48]. Undigested carbohydrates in stomach and small intestine can be fermented by microbes with the production of SCFAs in the large intestine [14]. SCFAs, including acetate, propionate, and butyrate, play an important role in the metabolic functions of the gut. In the present study, the *H. illucens* larvae induced an increase in the concentration of total SCFAs and butyrate. Previous studies also observed that *H. illucens* larvae as a soybean meal substitute increase the concentration of SCFAs in the cecum of laying hens [5, 11]. This increase may have been the result of an increased abundance or the number of microbes capable of producing butyrate in the colon. The correlation analysis indicated the trophic linkage between the abundance of *Roseburia*, *Pseudobutyrivibrio*, *Faecalibacterium*, the number of *Clostridium* cluster XIVa and butyrate concentration in the colonic digesta, suggesting that the increase in *Roseburia*, *Pseudobutyrivibrio*, *Faecalibacterium*, and *Clostridium* cluster XIVa may have resulted in the increase in colonic butyrate concentration. Additionally, *H. illucens* larvae are rich in chitin, which can not be degraded or absorbed by the small intestine but can flow into the large intestine, and subsequently be fermented by bacteria in large intestine. Thus, another potential explanation for the increase in butyrate concentration may be attributed to the alteration of substrate for microbial fermentation after administering *H. illucens* larvae. Butyrate is the major energy source for colonic epithelial cells and exerts an anti-inflammatory function [49]. Therefore, the increase in butyrate concentration in the current study suggests a host-friendly gut environment after administering *H. illucens* larvae.

### *H. illucens* larvae markedly decrease the end products of amino acid fermentation

Undigested protein or other nitrogenous compounds, such as amino acids, are fermented by microbes to produce BCFAs, biogenic amines, ammonia, phenol and indole compounds [14]. In the present study, *H. illucens* larvae markedly impacted the nitrogen metabolic profiles. For biogenic amines, cadaverine and putrescine are the most abundant amines and are formed from decarboxylation of lysine, arginine and ornithine, respectively [14]. A diet including 4% *H. illucens* larvae markedly decreased the concentrations of total amines and cadaverine compared with the control group, suggesting decreased microbial amino acid decarboxylation, which may be attributed to a decrease in the abundance of amines-producing bacteria in the colon. Our correlation analysis showed that the decreases in total amines and cadaverine were positively correalted with the decreased abundance of *Streptococcus*. This finding is consistent with some previous studies, showing that *Streptococcus* bacteria have amine-producing ability [48, 50]. High levels of biogenic amines, such as cadaverine, might be toxic to gut health [51]. Thus, the decrease in total amines and cadaverine concentrations by *H. illucens* larvae may extert a beneficial effect on gut health.

On the other hand, administering *H. illucens* larvae also altered the aromatic amino acid metabolism by bacteria in the colon. Tryptophan, an aromatic amino acid, can be decarboxylated into tryptamine by gut bacteria [13, 14]. In the present study, diet including 4% *H. illucens* larvae decreased the tryptamine concentration in the colonic digesta. Tryptamine induces serotonin secretion by enterochromaffin cells and subsequently regulates intestinal motility and platelet function [52]. Therefore, the decrease in tryptamine may affect intestinal function. In addition, *H. illucens* larvae also reduced the concentrations of phenol, *p*-cresol, and skatole, indicating that bacterial fermentation of tryptophan and tyrosine decreased. Phenol and *p*-cresol are produced from tyrosine catabolism by gut bacteria, while skatole is derived from tryptophan catabolism [14], and are regarded as co-carcinogens and colon cancer promoter [53]. Thus, the decrease in the concentrations of phenolic and indolic compounds in the current study indicates that *H. illucens* larvae may have a beneficial effect on gut health. In general, together with the increase in SCFAs, our findings clearly indicate an evident change in microbial metabolic activity, with higher microbial carbohydrate fermentation and lower microbial catabolism of amino acids after the *H. illucens* larvae treatment.

### *H. illucens* larvae affected the colonic mucosal response involved in colonic health

The change of intestinal microbiota and their metabolites could affect intestinal immunity of host. In the current study, diet including *H. illucens* larvae (especially supplementation with 4% group) down-regulated mRNA expression of *TLR4* and pro-inflammatory cytokines *IFN-γ*, while up-regulated the expression of anti-inflammatory cytokines *IL-10* in the colonic mucosa. As the novel receptors, TLR can mediate innate immune responses, recognize microbiota and their products and then initiate pro-inflammatory pathways [54]. Some species of *Streptococcus* [55] and their products (amines, indolic and phenolic compounds) [13, 14] can trigger intestinal inflammation through *TLR4* signaling pathways, while SCFAs-producing bacteria and SCFAs can down-regulate the pro-inflammatory responses through *TLR4-NFkB* signaling pathway [56]. In particular, the correlation analysis also revealed that the down-regulation in the expressions of *TLR4* and pro-inflammatory cytokines (*IFN-γ*) positively correlated with the decreased abundance of *Streptococcus* and the concentrations of total amines, cadaverine, and phenol, while negatively correlated with the increase in butyrate-producing bacteria (*Psedobutyrivibrio*, *Roseburia*, *Faecalibacterium*, *Clostridium* cluster XIVa) and the concentrations total SCFA, and butyrate (Fig. 7). Thus, the down-regulation gene expression levels of pro-inflammatory cytokines while up-regulation of the anti-inflammatory cytokine after the *H. illucens* larvae treatment may be attributed to enrichments of some butyrate-producing bacteria and SCFAs concentrations, and the depression of potential pathogen (*Streptococcus*) abundance and several toxic compounds concentrations.

As the critical line, the intestinal barrier can protect against pathogenic agents and luminal antigens. In the current study, dietary inclusion of *H. illucens* larvae up-regulated the expression of intestinal barrier genes expression in the colonic mucosa, such as *ZO-1*, *occludin*, and *mucin-1*, suggesting that *H. illucens* larvae may enhance the integrity of the mucous layer, and then generate a host-friendly gut environment which can defend against pathogen infection. To the best of our knowledge, our study is the first to test partially *H. illucens* larvae inclusion in the diets of finishing pigs, and the available information on the effect of dietary *H. illucens* larvae inclusion on mucosal response involved in colonic health is very limited. A previous study has shown that SCFAs, especially butyrate can maintain the gut integrity [56]. As mentioned above, diet inclusion of *H. illucens* larvae increased the concentrations of total SCFAs and butyrate, and correlation analysis also revealed that the total SCFAs and butyrate concentrations and SCFA-producing bacteria were positively correlated with mRNA expression levels of *ZO-1*, *occludin*, and *mucin-1*. Therefore, the high total SCFAs and butyrate concentrations and the abundance or counts of SCFA-producing bacteria after the *H. illucens* larvae treatment may lead to up-regulation of intestinal barrier genes expression, but further studies will be required to clarify the underlying mechanisms. Nonetheless, these findings suggest that diet inclusion of *H. illucens* larvae down-regulated the expression of pro- inflammatory genes, while up-regulated the expression of anti-inflammatory and intestinal barrier genes.

## Conclusion

In conclusion, the present study demonstrates that dietary supplementation with 4% *H. illucens* larvae meal significantly altered colonic microbial composition, metabolic profiles, and immune status of finishing pigs, likely toward a host-friendly gut environment. Colonic bacteria, such as *Lactobacillus* and several butyrate-producing bacteria were increased after *H. illucens* larvae supplementation, whereas the abundance of *Streptococcus* was decreased. Intestinal metabolism was altered by *H. illucens* larvae, as evidenced by the increase in the concentrations of SCFAs, and the decrease in the concentrations of metabolites involved in amino-acid metabolism. The colonic mucosal mRNA expression of *TLR-4* and pro-inflammatory cytokines were decreased, while the expression of anti-inflammatory and intestinal barrier genes were increased. Furthermore, the changes in the microbiota composition and metabolites concentrations were associated with the alterations in the colonic mucosal immune homeostasis and barrier function. These findings provide a new perspective on insect meal as a sustainable protein source rich in nutrients for swine feed.

## Additional files

Additional file 1: **Table S1.** Primers used for quantification in this study. (DOCX 16 kb)

Additional file 2: **Table S2.** Primers used for host genes in this study. (DOCX 15 kb)

Additional file 3: **Figure S1.** Rarefaction curves comparing the number of sequences with the number of phylotypes found in the 16S rRNA gene libraries from the microbiota in the digesta of the colon of pigs. (TIF 12962 kb)

Additional file 4: **Figure S2.** Influence of *Hermetia illucens* larvae meal on the 30 most abundant genera in the colonic digesta. The color represents the relative abundance of bacteria. (TIF 15073 kb)

Additional file 5: **Figure S3.** Spearman’s correlation analysis between the abundance of colonic microbiota (at the genus level and qPCR) and microbial metabolites affected by dietary treatment. Cells are colored based on the correlation coefficient between the significantly changed bacteria (the relative abundance and the numbers of bacteria) and metabolites (concentrations). The intensity of the colors represents the degree of association. Red represented a significant positive correlation (*P* < 0.05), blue represents significantly negative correlation (*P* < 0.05), and white shows that the correlation was not significant (*P* > 0.05). Total SCFA: total short-chain fatty acids. (TIF 9517 kb)

## Abbreviations

AMOVA: Analysis of molecular variance
BCFA: Branched-chain fatty acid
FDR: False discovery rate
IFN-γ: Interferon-γ
IL: Interleukin
OTU: Operational taxonomic units
PCoA: Principal coordinate analysis
**S**CFAs: Short-chain fatty acids
TLR: Toll-like receptors
TNF-α: Tumor necrosis factor-α
ZO-1: Zona occludens protein 1
