# Supplementary material for: Hermetia illucens larvae as a potential dietary protein source altered the microbiota and modulated mucosal immune status in the colon of finishing pigs
Source: J Anim Sci Biotechnol. 2019 Jun 19;10:50. doi: 10.1186/s40104-019-0358-1 (PMC6582608; doi:10.1186/s40104-019-0358-1)
Supplement: Supplementary file 1 — Table S1. Primers used for quantification in this study. (DOCX 16 kb) [file 40104_2019_358_MOESM1_ESM.docx]

**Additional files 1**

**Supplementary Table S1.** Primers used for quantification in this study

| Target | Primer sequence 5′-3′ | Reference |
| --- | --- | --- |
| Total bacteria | Forward: CGGTGAATACGTTCYCGG  Reverse: GGWTACCTTGTTACGACTT | [1] |
| Firmicutes | Forward: GGAGYATGTGGTTTAATTCGAAGCA | [2] |
|  | Reverse: AGCTGACGACAACCATGCAC |  |
| Bacteroidetes | Forward: GGARCATGTGGTTTAATTCGATGAT | [2] |
|  | Reverse: AGCTGACGACAACCATGCAG |  |
| *Clostridium* cluster IV | Forward: GCACAAGCAGTGGAGT | [3] |
|  | Reverse: CTTCCTCCGTTTTGTCAA |  |
| *Clostridium* cluster XIVa | Forward: CGGTACCTGACTAAGAAGC | [4] |
|  | Reverse: AGTTTYATTCTTGCGAACG |  |
| *Escherichia.coli* | Forward: CATGCCGCGTGTATGAAGAA | [5] |
|  | Reverse: CGGGTAACGTCAATGAGCAAA |  |
| *Bifidobacterium* | Forward: TCGCGTCYGGTGTGAAAG | [6] |
|  | Reverse: GGTGTTCTTCCCGATATCTAC |  |
| *Lactobacillus* | Forward: AGCAGTAGGGAATCTTCCA | [7] |
|  | Reverse: ATTCCACCGCTACACATG |  |
| *Bacteroides-Prevotella* | Forward: GAGAGGAAGGTCCCCCAC | [8] |
|  | Reverse: CGCTACTTGGCTGGTTCAG |  |
| *Ruminococcus* | Forward: GAAAGCGTGGGGAGCAAACAGG | [9] |
|  | Reverse: GACGACAACCATGCACCACCTG |  |

**Reference:**

1. Suzuki M T, Taylor LT, DeLong EF. Quantitative analysis of small-subunit rRNA genes in mixed microbial populations via 5′-nuclease assays. Appl Environ Microbiol 2000;66 (11):4605-14.

2. Guo X, Xia X, Tang R, Zhou J, Zhao H, Wang K. Development of a real‐time PCR method for Firmicutes and Bacteroidetes in faeces and its application to quantify intestinal population of obese and lean pigs. Letters Appl Microbiol. 2008; 47(5):367-73.

3. Matsuki T, Watanabe K, Fujimoto J, Takada T, Tanaka R. Use of 16S rRNA gene-targeted group-specific primers for real-time PCR analysis of predominant bacteria in human feces. Appl Environm Microbiol.2004;*70* (12): 7220-28.

4. Bartosch S, Fite A, Macfarlane GT, McMurdo ME.Characterization of bacterial communities in feces from healthy elderly volunteers and hospitalized elderly patients by using real-time PCR and effects of antibiotic treatment on the fecal microbiota. Appl Environm Microbiol. 2004; 70 (6):3575-81.

5. Huijsdens XW, Linskens RK, Mak M, Meuwissen S G, Vandenbroucke-Grauls CM, Savelkoul P H. Quantification of bacteria adherent to gastrointestinal mucosa by real-time PCR. J Clin Microbiol. 2002;40 (12):4423-27.

6. Walker AW, Ince J, Duncan SH, Webster LM, Holtrop G, Ze X, Brown D, Stares MD, Bergerat A. Dominant and diet-responsive groups of bacteria within the human colonic microbiota. ISME J. 2011;*5* (2):220-30.

7. Khafipour E, Li S, Plaizier JC, Krause DO.,Rumen microbiome composition determined using two nutritional models of subacute ruminal acidosis. Appl Environm Microbiol. 2009; 75 (22):7115-24.

8. Layton A, Mckay L, Dan W, Garrett V, Gentry R, Sayler G.Development of bacteroides 16S rRNA gene TaqMan-Based real-time PCR assays for estimation of total, human, and bovine fecal pollution in water. Appl Environ Microbiol. 2006;*72* (6):4214-24.

9. Verma R, Verma A K, Ahuja V, Paul J. Real-time analysis of mucosal flora in patients with inflammatory bowel disease in India. J Clin Microbiol 2010, 48 (11), 4279.
