# Supplementary material for: Hermetia illucens larvae as a potential dietary protein source altered the microbiota and modulated mucosal immune status in the colon of finishing pigs
Source: J Anim Sci Biotechnol. 2019 Jun 19;10:50. doi: 10.1186/s40104-019-0358-1 (PMC6582608; doi:10.1186/s40104-019-0358-1)
Supplement: Supplementary file 2 — Table S2. Primers used for host genes in this study. (DOCX 15 kb) [file 40104_2019_358_MOESM2_ESM.docx]

**Additional files 2**

**Table S2.** Primers used for host genes in this study

| Target | Primer sequence 5′-3′ | Reference |
| --- | --- | --- |
| Beta actin | Forward: AGAGCGCAAGTACTCCGTGT | [[1](#_ENREF_10)] |
|  | Reverse: ACATCTGCTGGAAGGTGGAC |  |
| *TLR2* | Forward: TCACTTGTCTAACTTATCATCCTCTTG | [[2](#_ENREF_11)] |
|  | Reverse: TCAGCGAAGGTGTCATTATTGC |  |
| *TLR4* | Forward: TCAGTTCTCACCTTCCTCCTG | [[3](#_ENREF_12)] |
|  | Reverse: GTTCATTCCTCACCCAGTCTTC |  |
| *TLR5* | Forward: CAGCGACCAAAACAGATTGA | [[2](#_ENREF_11)] |
|  | Reverse: TGCTCACCAGACAGACAACC |  |
| *IL-8* | Forward: ACTGGCTGTTGCCTTCTT | [[4](#_ENREF_13)] |
|  | Reverse: CAGTTCTCTTCAAAAATATCTG |  |
| *IL-10* | Forward: GTCCGACTCAACGAAGAAGG | [1] |
|  | Reverse: GCCAGGAAGATCAGGCAATA |  |
| *IFN-γ* | Forward: TCCAGCGCAAAGCCATCAGTG | [5] |
|  | Reverse: ATGCTCTCTGGCCTTGGAACATAGT |  |
| *TNF-α* | Forward: CCACGCTCTTCTGCCTACTGC | [4] |
|  | Reverse: GCTGTCCCTCGGCTTTGAC |  |
| *Mucin-1* | Forward: GGTACCCGGCTGGGGCATTG | [1] |
|  | Reverse: GGTAGGCATCCCGGGTCGGA |  |
| *Mucin-2* | Forward: CTGCTCCGGGTCCTGTGGGA | [1] |
|  | Reverse: CCCGCTGGCTGGTGCGATAC |  |
| *Occludin* | Forward: ATGCTTTCTCAGCCAGCGTA | [4] |
|  | Reverse: AAGGTTCCATAGCCTCGGTC |  |
| *ZO-1* | Forward: GAGGATGGTCACACCGTGGT | [4] |
|  | Reverse: GGAGGATGCTGTTGTCTCGG |  |

**Reference:**

1. Pieper R, Kröger S, Richter JF, Wang J, Martin L, Bindelle J, Htoo JK, Von SD, Vahjen W, Zentek J. Fermentable fiber ameliorates fermentable protein-induced changes in microbial ecology, but not the mucosal response, in the colon of piglets. J Nutr. 2012; 142 (4), 661-67.

2. Collado-Romero M, Arce C, Ramírez-Boo M, Carvajal A, Garrido JJ. Quantitative analysis of the immune response upon *Salmonella typhimurium* infection along the porcine intestinal gut. Vet Res. 2010;41(2)23.

3. Yulan L, Feng C, Jack O, Xi L, Jacobi SK, Huiling Z, Zhifeng W, Yongqing H. Fish oil enhances intestinal integrity and inhibits TLR4 and NOD2 signaling pathways in weaned pigs after LPS challenge. J Nutr. 2012;*142* (11):2017-24.

4. Zhou XL, Kong XF, Lian GQ, Blachier F, Geng MM,Yin YL. Dietary supplementation with soybean oligosaccharides increases short-chain fatty acids but decreases protein-derived catabolites in the intestinal luminal content of weaned Huanjiang mini-piglets. Nutr Res. 2014;34 (9):780-88.

5. Carmen VT, Christelle B, Friederike S, Aschenbach JRR, Wilfried V, Jürgen Z, Robert P. Down-regulation of monocarboxylate transporter 1 (MCT1) gene expression in the colon of piglets is linked to bacterial protein fermentation and pro-inflammatory cytokine-mediated signalling. Brit J Nutr. 2015;113 (4):610-17.
