# Supplementary figures and images for: Hermetia illucens larvae as a potential dietary protein source altered the microbiota and modulated mucosal immune status in the colon of finishing pigs
Source: J Anim Sci Biotechnol. 2019 Jun 19;10:50. doi: 10.1186/s40104-019-0358-1 (PMC6582608; doi:10.1186/s40104-019-0358-1)

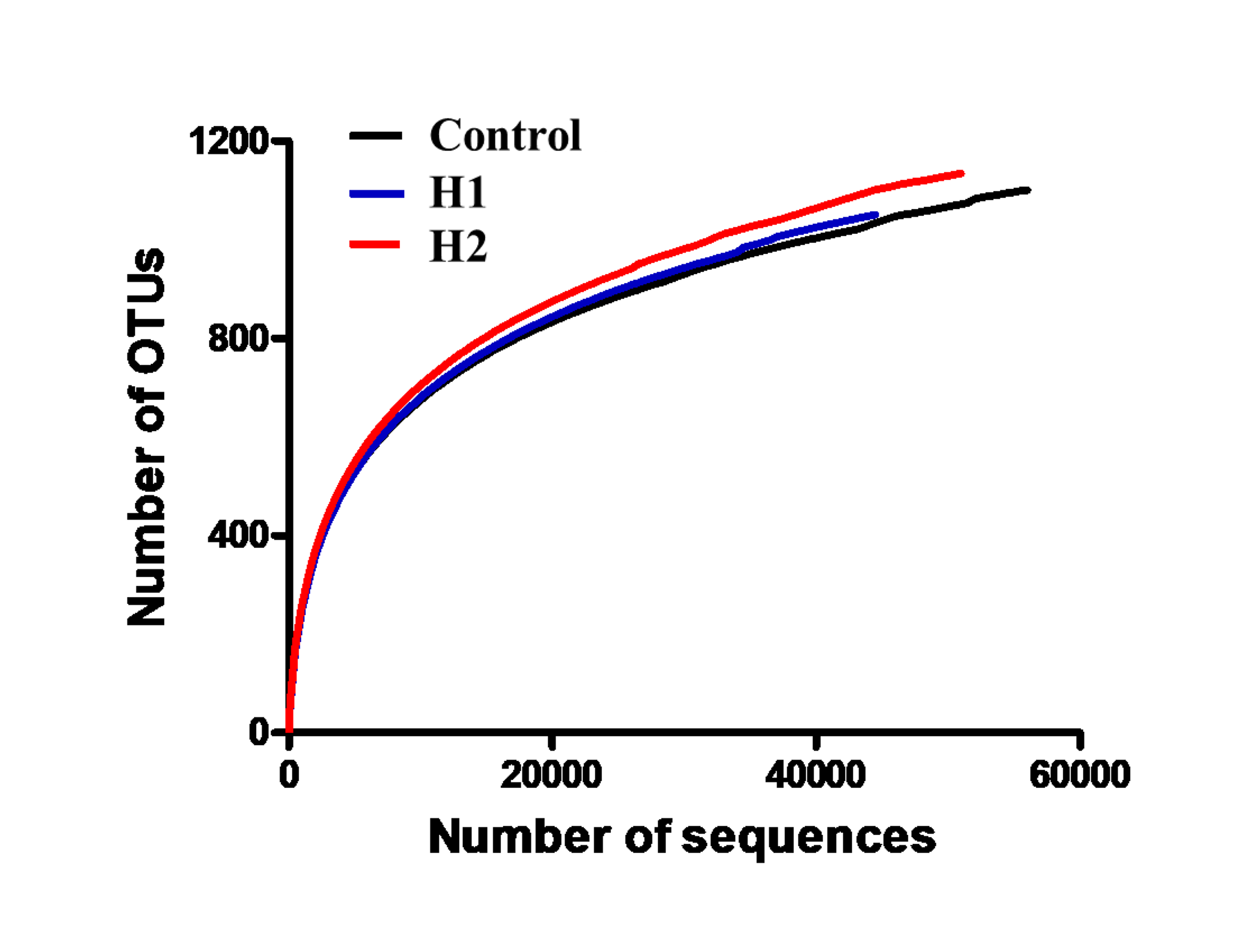

Supplement: Supplementary file 3 — Figure S1. Rarefaction curves comparing the number of sequences with the number of phylotypes found in the 16S rRNA gene libraries from the microbiota in the digesta of the colon of pigs. (TIF 12962 kb) [file 40104_2019_358_MOESM3_ESM.tif]

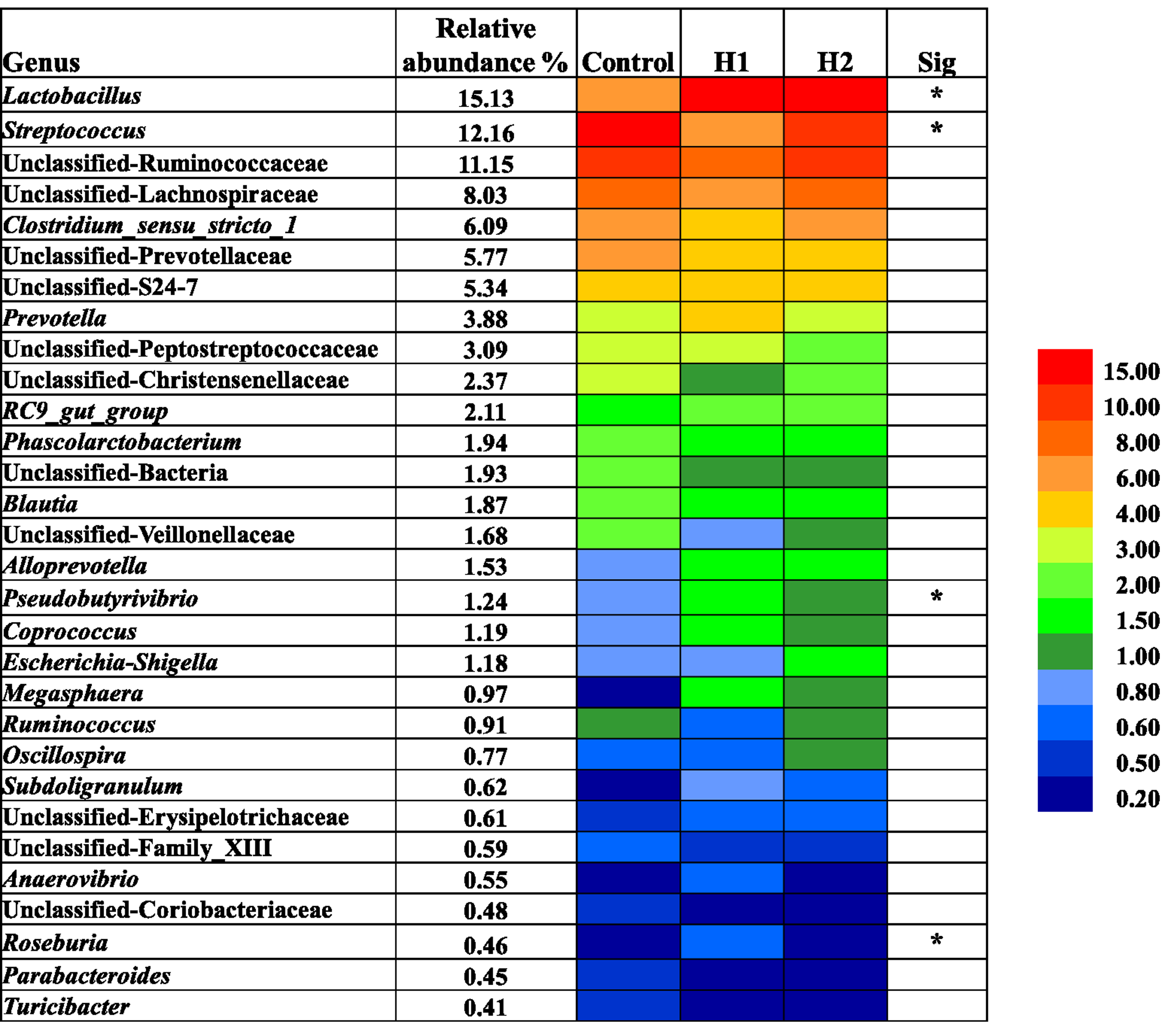

Supplement: Supplementary file 4 — Figure S2. Influence of Hermetia illucens larvae meal on the 30 most abundant genera in the colonic digesta. The color represents the relative abundance of bacteria. (TIF 15073 kb) [file 40104_2019_358_MOESM4_ESM.tif]

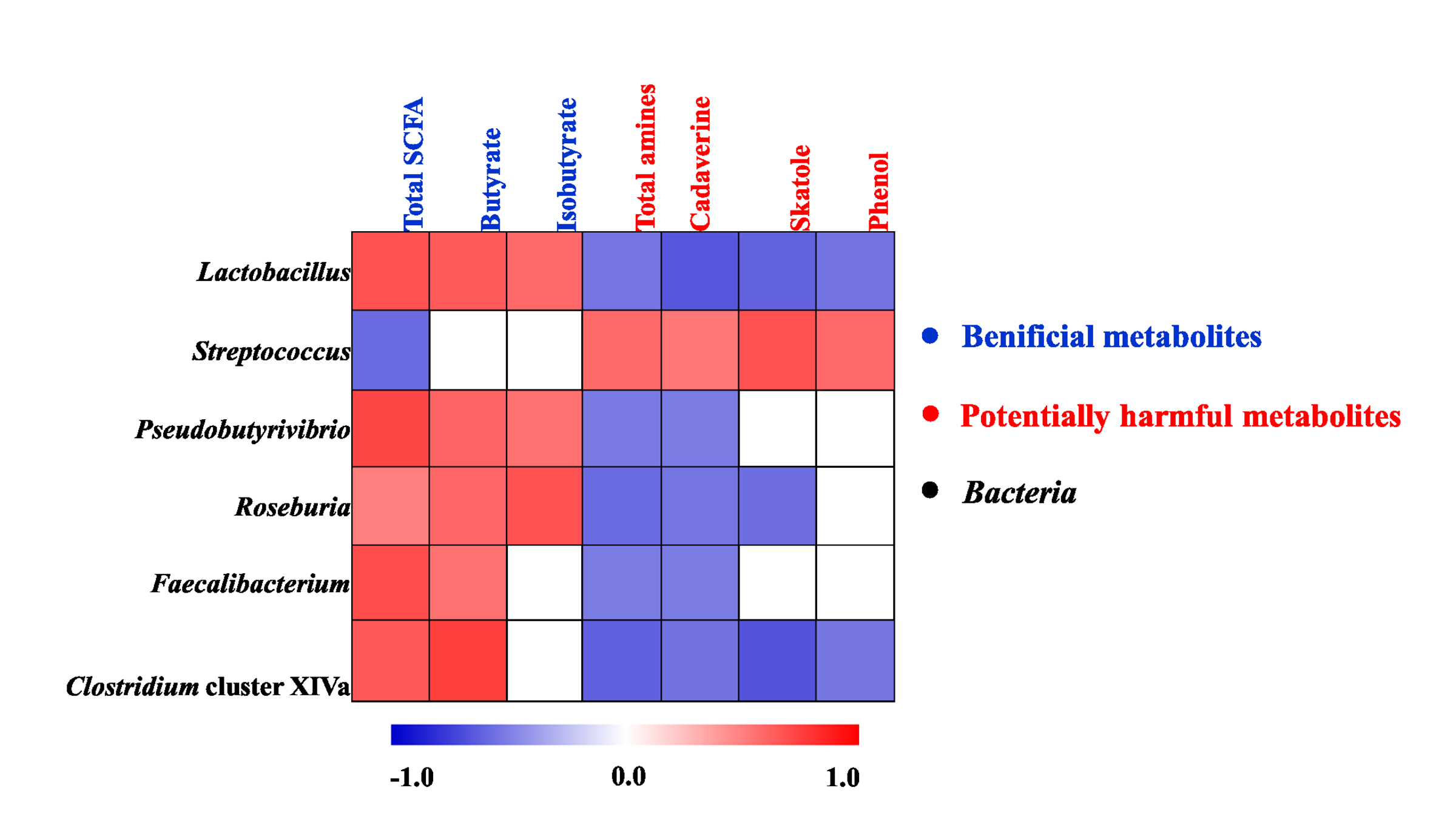

Supplement: Supplementary file 5 — Figure S3. Spearman’s correlation analysis between the abundance of colonic microbiota (at the genus level and qPCR) and microbial metabolites affected by dietary treatment. Cells are colored based on the correlation coefficient between the significantly changed bacteria (the relative abundance and the numbers of bacteria) and metabolites (concentrations). The intensity of the colors represents the degree of association. Red represented a significant positive correlation (P < 0.05), blue represents significantly negative correlation (P < 0.05), and white shows that the correlation was not significant (P > 0.05). Total SCFA: total short-chain fatty acids. (TIF 9517 kb) [file 40104_2019_358_MOESM5_ESM.tif]
